# The reproduction number and its probability distribution for stochastic viral dynamics

**DOI:** 10.1098/rsif.2023.0400

**Published:** 2024-01-24

**Authors:** Bevelynn Williams, Jonathan Carruthers, Joseph J. Gillard, Grant Lythe, Alan S. Perelson, Ruy M. Ribeiro, Carmen Molina-París, Martín López-García

**Affiliations:** ^1^ Department of Applied Mathematics, School of Mathematics, University of Leeds, Leeds, UK; ^2^ UK Health Security Agency, Salisbury, UK; ^3^ CBR Division, Defence Science and Technology Laboratory, Salisbury, UK; ^4^ T-6, Theoretical Biology and Biophysics, Theoretical Division, Los Alamos National Laboratory, Los Alamos, NM, USA

**Keywords:** viral burst size, reproduction number, eclipse phase, infectious phase, Erlang distribution, stochastic model

## Abstract

We consider stochastic models of individual infected cells. The reproduction number, *R*, is understood as a random variable representing the number of new cells infected by one initial infected cell in an otherwise susceptible (target cell) population. Variability in *R* results partly from heterogeneity in the viral burst size (the number of viral progeny generated from an infected cell during its lifetime), which depends on the distribution of cellular lifetimes and on the mechanism of virion release. We analyse viral dynamics models with an eclipse phase: the period of time after a cell is infected but before it is capable of releasing virions. The duration of the eclipse, or the subsequent infectious, phase is non-exponential, but composed of stages. We derive the probability distribution of the reproduction number for these viral dynamics models, and show it is a negative binomial distribution in the case of constant viral release from infectious cells, and under the assumption of an excess of target cells. In a deterministic model, the ultimate in-host establishment or extinction of the viral infection depends entirely on whether the mean reproduction number is greater than, or less than, one, respectively. Here, the probability of extinction is determined by the probability distribution of *R*, not simply its mean value. In particular, we show that in some cases the probability of infection is not an increasing function of the mean reproduction number.

## 1. Introduction

Viruses, and some types of bacteria, infect host cells. When a viral particle is taken up by a host cell, the genome is replicated and used to produce viral proteins. New viral particles are then assembled inside the infected cell. For some viruses, progeny virions accumulate inside the host cell and eventually numerous viral particles exit at once in a burst, killing the cell. On the other hand, for most enveloped viruses, new viral particles are released throughout the lifetime of the infected cell via a process called budding. The total number of virions released by an infected cell during its lifetime is usually referred to as the ‘burst size’, even if the virus is released continuously by budding [1–4].

Stochasticity of the intracellular viral life cycle, coupled with heterogeneity in the lifetime of infected cells, can lead to variability in the number of virions released by individual cells. For instance, Hataye *et al.* [5] measured burst sizes from HIV-1-infected cells, and Bacsik *et al.* [6] measured production from individual influenza A virus-infected cells, with both references encountering high variability. The progeny virions released by an infected cell may infect further host target cells, or may be degraded or cleared before they manage to do so. Hence, the number of secondary infected cells caused by a single infected cell will depend on its burst size and on the fraction of these progeny virions that are able to infect other cells.

The basic reproduction number was originally introduced in an epidemiological context to characterize the mean number of secondary infections caused by an initial infected individual in an otherwise susceptible population. Here we consider the cell-level equivalent in the context of within-host and *in vitro* viral dynamics, where the cell-level basic reproduction number will be understood as a random variable, *R*, representing the number of target cells infected by one initial infected cell in an otherwise susceptible (target) cell population [1,7–10]. The mean value of the basic reproduction number will be denoted by $\bar{R}$.

In deterministic models, the mean value of the basic reproduction number determines the outcome of infection: if $\bar{R}<1$, then the virus-free steady state is stable and the infection will die out. In stochastic models, if the mean number of secondary infections caused by a single infected cell is smaller than one, the infection can grow, but will certainly die out eventually. Conversely, when $\bar{R}>1$, deterministic models predict that an infection will be established, but in stochastic models there can be a non-zero probability that all virions and infected cells will be eliminated before the infection can become established [1,11]. This probability depends both on $\bar{R}$ [12], and on the probability distribution of the reproduction number [1,11]. By exclusively focusing on the mean value of the reproduction number, one may fail to capture important dynamics of early infection events, which are inherently stochastic, particularly if an individual is exposed to a low infecting viral dose.

We study two Markov chain viral infection models, with non-exponential infectious period distributions. The models are based on one of the simplest deterministic models of viral infection, consisting of a system of ODEs for the populations of target cells, infected cells and virions [13]. One model considers viral release by budding and the other by bursting. In the model of viral release by budding, productively infected (i.e. infectious) cells are assumed to release virions at a constant rate. In the model of viral release by bursting, viral particles are produced intracellularly at a constant rate, and are eventually released in a burst when the infected cell dies. Pearson *et al.* [1] studied stochastic models similar to these, with a single infected cell compartment. We split the infected cell state into multiple compartments, leading to an Erlang-distributed, instead of an exponentially distributed, infectious period [2,7,14,15]. An Erlang distribution may be a more suitable characterization of the infectious period than an exponential one, and can lead to different estimates of model parameters [16]. The exponential case can still be recovered by setting the shape parameter of the Erlang distribution (given by the number of infected cell compartments) to one.

The deterministic model, and the starting point for our stochastic models, is successful in describing viral infections when the populations of infected cells and virions are large, and has been used to estimate viral kinetic parameters in the literature. On the other hand, the reproduction number probability distribution is particularly important to characterize the early stages of viral infection, when there may only be a few extracellular virions or infected cells. Even if the mean reproduction number is fairly large, the initial infection may die out by chance, due to a non-zero probability that an infected cell will cause no secondary infections. This highlights the importance of looking beyond the mean of the reproduction number, and of considering other summary statistics from the probability distribution of this random variable. Previous studies that have focused on calculating the probability of viral extinction during the early stages of infection include Pearson *et al.* [1], who calculated the probability of extinction in models with either a geometric or Poisson distribution of the reproduction number. In this paper, we aim to show that deriving and quantifying the burst size and reproduction number distributions for different models and viruses can help to better understand how these distributions are affected by modelling choices and parameters, and how these distributions, in turn, affect the probability that the virus and infected cells will be eliminated before an infection becomes established.

Section 2 contains model descriptions, and derivations of the probability distributions of the burst size and reproduction number for these models. In §3, we obtain numerical results for the reproduction number probability distribution and the probability of viral extinction, in order to investigate the effects of various model assumptions and parameter values on these quantities of interest. In the electronic supplementary material, we show that similar results can be obtained for models with an age-dependent viral release rate.

## 2. Mathematical methods

We begin with models of viral dynamics described by ordinary differential equations (ODEs) in §2.1. We introduce a model in which infectious cells produce and release virions via budding at a constant rate, and we also consider a model in which virions are instead released in a burst. We then describe the Markov chain versions of these models in §2.2. For each model, we derive the average burst size and reproduction number (§2.3), the probability distributions of the burst size and reproduction number (§2.4 and §2.5) and the probability of viral extinction (§2.6). In the electronic supplementary material, we consider a model developed by Guedj *et al.* [17] for hepatitis C virus (HCV) infection. This model considers viral release by budding, but includes intracellular dynamics, resulting in a viral release rate from infected cells that is not constant but depends on the intracellular viral genome counts. We derive the probability distributions of the burst size and reproduction number for a stochastic version of this model, and make use of the parameter estimates from Guedj *et al.* [17] to obtain numerical results.

### 2.1. Deterministic models

#### 2.1.1. Viral release by budding

**Figure 1.**
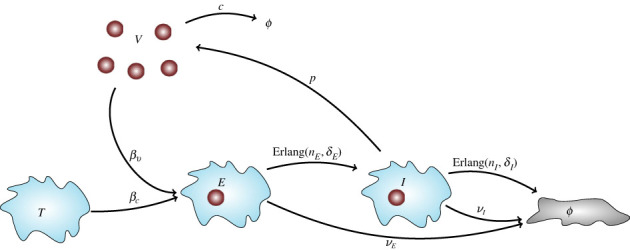
Model with constant viral release rate. Upon infection by a free (i.e. extracellular) infectious virion *V*, a target cell, *T*, enters the eclipse phase *E* followed by the infectious phase *I*. The arrows show the possible transitions and their corresponding rates, or the distribution of times, in the case of non-exponentially (Erlang) distributed transition times. Free virions are cleared with rate *c*, and eclipse-phase cells with rate *ν*_*E*_. Infectious cells are cleared by the immune system with rate *ν*_*I*_, or undergo virus-induced cell death. In (2.1), the eclipse and infectious phases consist of *n*_*E*_ and *n*_*I*_ stages, respectively.

Our ODE model of viral release by budding is based on those of Liao *et al.* [2] and Yan *et al.* [7], with a modification to represent removal of infected cells by the immune system [11,18]. Uninfected target cells are infected by viral particles with (per virion) rate *β*_*c*_, then pass through an eclipse phase and an infectious phase. Thus, the eclipse phase is the period after viral entry but before the release of virions. The population of infected cells is partitioned into *n*_*E*_ + *n*_*I*_ subsets: *n*_*E*_ subsets *E*_*i*_, one for each eclipse-phase stage, and *n*_*I*_ subsets *I*_*i*_, one for each infectious-phase stage. A cell in any of the infectious-phase stages releases virions at constant rate *p*. It dies upon exiting the last infectious stage. A diagram is provided in figure 1. The system of 2 + *n*_*E*_ + *n*_*I*_ ODEs is

2.1 $$\begin{array}{rl} & \frac{dT}{dt}=-\beta_{c}TV,\\ & \frac{dE_{1}}{dt}=\beta_{c}TV-\left(\frac{n_{E}}{\tau_{E}}+\nu_{E}\right)E_{1},\\ & \frac{dE_{i}}{dt}=\frac{n_{E}}{\tau_{E}}(E_{i-1}-E_{i})-\nu_{E}E_{i},\;i=2,\ldots ,n_{E},\\ & \frac{dI_{1}}{dt}=\frac{n_{E}}{\tau_{E}}E_{n_{E}}-\left(\frac{n_{I}}{\tau_{I}}+\nu_{I}\right)I_{1},\\ & \frac{dI_{i}}{dt}=\frac{n_{I}}{\tau_{I}}(I_{i-1}-I_{i})-\nu_{I}I_{i},\;i=2,\ldots ,n_{I},\\ \; & \frac{dV}{dt}=p\sum_{i=1}^{n_{I}}I_{i}-cV-\beta_{v}TV. \end{array}$$

The variables *T* and *V* denote the number of uninfected (or susceptible) target cells, and the number of free (i.e. extracellular) infectious virions. In the first ODE, some models also include production and death of uninfected target cells. However in many circumstances, these events are neglected when considering a short timescale [1]. Examples are found in models describing *in vitro* viral dynamics [2,7] or *in vivo* acute infection dynamics, where the viral load increases to a maximum, and then declines due to the depletion of target cells. Thus, we do not consider the generation of new target cells or death of uninfected target cells in our model.

The multi-stage representation of the eclipse and infectious phases in (2.1) is equivalent to the assumption that the time a cell spends in either phase follows an Erlang distribution. The Erlang distribution results from a sum of exponential random variables, and is a special case of the gamma distribution, with an integer-valued shape parameter. Here, the mean time spent in the eclipse phase (with *n*_*E*_ stages) is *τ*_*E*_, so the mean time spent in each eclipse-phase stage is 1/*δ*_*E*_ = *τ*_*E*_/*n*_*E*_. The mean time spent in the infectious phase (with *n*_*I*_ stages) is *τ*_*I*_, so the mean time spent in each infectious-phase stage is 1/*δ*_*I*_ = *τ*_*I*_/*n*_*I*_.

An additional mechanism of infected cell death due to the immune response, even before they release virions, has been included in the model by specifying that eclipse-phase cells are cleared with rate *ν*_*E*_, and infectious-phase cells with rate *ν*_*I*_ [18]. Eclipse-phase cells have been found to express viral peptides, so CD8^+^ T cells can recognize and kill them [19,20]. If eclipse-phase cells are killed at a slower rate than infectious-phase ones then *ν*_*E*_ < *ν*_*I*_. The model without immune response can be recovered by setting *ν*_*E*_ = *ν*_*I*_ = 0, for instance, in order to represent *in vitro* dynamics [2,7]. When *ν*_*E*_ = *ν*_*I*_ = 0, the duration of the eclipse and infectious phases follow Erlang distributions. However, when *ν*_*E*_ > 0 and *ν*_*I*_ > 0, the distributions are altered by the possibility of cell death due to the immune response; that is, the contingency that the eclipse or infectious phase ‘completes early’ due to the cell being killed by the immune response.

Some of the virions released by infectious cells are non-infectious and cannot, therefore, infect new target cells. Here we assume that *p* denotes the release rate of *infectious* virions, and that the variable *V* represents the number of *infectious* viral particles. Free viral particles are cleared with rate *c*, which can be thought of as the sum of the rates of viral degradation and of loss due to antibody binding. In the last ODE, the term *β*_*v*_ *TV* corresponding to loss of virions due to infection is explicitly included, although it is often neglected in the literature based on being small compared with the term *cV* [21]. Furthermore, if the number of target cells is assumed to remain constant, *T*_0_, both viral loss terms can be incorporated into one parameter, *c*′ = *c* + *β*_*v*_ *T*_0_ [22].

The parameters *β*_*c*_ and *β*_*v*_ in (2.1) have different dimensions: *β*_*c*_ has dimensions of per virion per time, whereas *β*_*v*_ has dimensions of per cell per time. Let us introduce *β*, the rate at which infection events occur, with units of per cell per virion per time. If *n*_*c*_ and *n*_*v*_ are the number of cells and virions involved in a general infection event, respectively, then *β*_*c*_ = *n*_*c*_*β* and *β*_*v*_ = *n*_*v*_*β*. Here, since an infection event in the model consists of one virion infecting one target cell, we have *n*_*c*_ = *n*_*v*_ = 1. Therefore, numerically we have *β*_*c*_ = *β*_*v*_ = *β*, and from now on we will simply denote both rates by *β*.

An important quantity that can be calculated from (2.1), sometimes called the basic reproduction number, is the mean number of infected cells produced by a single infected cell. When *ν*_*E*_ = *ν*_*I*_ = 0, the mean number of virions released by a single infected cell is *pτ*_*I*_. If the number of target cells present is *T*_0_, then each of the released virions infects a target cell with probability *βT*_0_/(*c* + *βT*_0_). Provided *T*_0_ is large, the mean number of cells infected by virions released from a single infected cell is

2.2 $$\bar{R}=\frac{\;p\tau_{I}\beta T_{0}}{c+\beta T_{0}}.$$

For $\bar{R}$ as defined in (2.2), in the limit *T*_0_ → +∞, we have $\bar{R}\to p\tau_{I}$, which is the mean number of virions released from an infected cell. However, for models that neglect the ODE term corresponding to loss of virions due to infection [2], the mean reproduction number is

$$\bar{R}=\frac{\;p\tau_{I}\beta T_{0}}{c},$$

which becomes infinite as *T*_0_ → +∞. This implies that there is no finite bound on the mean number of secondary infections produced by an infected cell, so that as the initial target cell population becomes larger, the mean number of secondary infections will eventually become larger than the mean number of virions released from a single infected cell. This is not reasonable since each virion can only infect at most one cell.

#### 2.1.2. Viral release by bursting

In the model (2.1), viral particles are released from infectious cells by budding. However, for some viruses, progeny virions accumulate inside an infected host cell and are released in a burst upon cell lysis. Yuan and Allen considered a model of viral dynamics with bursting and included a mechanism to represent the immune response [11]. They made the assumption that if the death of an infected cell is virus-induced, then virions will be released in a burst, but if the infected cell is killed due to the action of an immune response, no virions will be released. Yuan and Allen [11] also assumed that each burst event leads to the same burst size. That is, if an infected cell bursts, its burst size would always be *N* = *pτ*_*I*_ and would not depend on the time when the cell bursts. This might be the case if the accumulation of virions inside the cell and the burst of the cell are coupled in such a way that the burst of the cell takes place when the number of intracellular virions reaches a given threshold. By contrast, here we consider a case in which the accumulation of virions and the burst of the cell are independent processes. In this case, cells which burst earlier may not produce as many viral particles as those that survive longer. This introduces variability in the burst size, related to the times of the burst events. We assume that intracellular virions are produced at a constant rate, *p*, during the ‘infectious’ phase, but are not released unless the cell bursts. We note that under this assumption, the eclipse phase represents a period in which the cell begins to synthesize viral proteins and to replicate the viral genome, but progeny virions have not yet started to be assembled.

In order to describe these events with an ODE model, we require additional variables to represent the total number of intracellular virions in the population of infectious cells. We let the variable *I*_*i*_ denote the number of infected cells in stage *i*, and the variable *P*_*i*_ denote the total number of intracellular infectious virions in all infected cells of stage *i*. In a given infected cell, intracellular virions are assumed to be produced at a constant rate, *p*. Each *P*_*i*_ increases due to virion production at a rate proportional to the number of cells in infectious stage *i* and due to cells entering stage *i*. *P*_*i*_ decreases when cells exit stage *i*. The rate at which a cell in stage *i* will exit this stage (either by transitioning to stage *i* + 1 or due to immune clearance) is (*δ*_*I*_ + *ν*_*I*_)*I*_*i*_. In the deterministic model, the *P*_*i*_ virions are equally shared between all cells of stage *i*. Therefore, the number of virions that will be cleared or will transition to the next stage when such an event happens will be *P*_*i*_/*I*_*i*_. Thus, the overall rate at which virions transition from compartment *P*_*i*_ to *P*_*i*+1_ (or get released in a burst for *i* = *n*_*I*_) is $\delta_{I}I_{i}\frac{P_{i}}{I_{i}}=\delta_{I}P_{i}$. The system of 2 + *n*_*E*_ + 2*n*_*I*_ ODEs is

2.3 $$\begin{array}{rl} & \frac{dT}{dt}=-\beta_{c}TV,\\ & \frac{dE_{1}}{dt}=\beta_{c}TV-(\delta_{E}+\nu_{E})\;E_{1},\\ & \frac{dE_{i}}{dt}=\delta_{E}(E_{i-1}-E_{i})-\nu_{E}E_{i},\;i=2,\ldots ,n_{E},\\ & \frac{dI_{1}}{dt}=\delta_{E}E_{n_{E}}-(\delta_{I}+\nu_{I})\;I_{1},\\ & \frac{dI_{i}}{dt}=\delta_{I}(I_{i-1}-I_{i})-\nu_{I}I_{i},\;i=2,\ldots ,n_{I},\\ & \frac{dP_{1}}{dt}=pI_{1}-(\delta_{I}+\nu_{I})\;P_{1},\\ & \frac{dP_{i}}{dt}=pI_{i}+\delta_{I}(P_{i-1}-P_{i})-\nu_{I}P_{i},\;i=2,\ldots ,n_{I},\\ \; & \frac{dV}{dt}=\delta_{I}P_{n_{I}}-cV-\beta_{v}TV. \end{array}$$

### 2.2. Stochastic models

We will consider stochastic versions of the models in (2.1) and (2.3), to then compute the associated probability distributions of the burst size and reproduction number.

#### 2.2.1. Viral release by budding

The model in (2.1) can be formulated as a continuous-time Markov chain (CTMC), X={X(t) : t∈[0,+∞)}, where

$$X(t)=(V(t),T(t),E_{1}(t),\ldots ,E_{n_{E}}(t),I_{1}(t),\ldots ,I_{n_{I}}(t)).$$

*V*(*t*), *T*(*t*), *E*_*i*_(*t*) and *I*_*j*_(*t*) (for *i* ∈ {1, …, *n*_*E*_} and *j* ∈ {1, …, *n*_*I*_}) are discrete random variables with values in the set of non-negative integers, for *t* ∈ [0, +∞). The possible transitions allowed in the stochastic model are shown in table 1.

**Table 1.** Transitions and their corresponding rates in the Markov chain version of the model in (2.1). We abuse notation and denote by (V(t),T(t), $E_{1}(t),\ldots ,E_{n_{E}}(t),I_{1}(t),\ldots ,I_{n_{I}}(t))$ the random variables of the stochastic process, while $\left(V,T,E_{1},\ldots ,E_{n_{E}},I_{1},\ldots ,I_{n_{I}}\right)$ represents a state of the process at any given time. Only the variables that change in a given transition are included in the middle column.

event	state transition			rate
infection	(*V*, *T*, *E*_1_)	⟶	(*V* − 1, *T* − 1, *E*_1_ + 1)	*βTV*
eclipse stage progression	(*E*_*i*_, *E*_*i*+1_)	⟶	(*E*_*i*_ − 1, *E*_*i*+1_ + 1)	*δ*_*E*_*E*_*i*_
immune system killing	*E*_*i*_	⟶	*E*_*i*_ − 1	*ν*_*E*_*E*_*i*_
eclipse to infectious phase	$\left(E_{n_{E}},I_{1}\right)$	⟶	$\left(E_{n_{E}}-1,I_{1}+1\right)$	$\delta_{E}E_{n_{E}}$
infectious stage progression	(*I*_*i*_, *I*_*i*+1_)	⟶	(*I*_*i*_ − 1, *I*_*i*+1_ + 1)	*δ*_*I*_*I*_*i*_
immune system killing	*I*_*i*_	⟶	*I*_*i*_ − 1	*ν*_*I*_*I*_*i*_
death of infectious cell	$I_{n_{I}}$	⟶	$I_{n_{I}}-1$	$(\delta_{I}+\nu_{I})I_{n_{I}}$
viral release by budding	*V*	⟶	*V* + 1	$p\sum_{i=1}^{n_{I}}I_{i}$
loss of extracellular virions	*V*	⟶	*V* − 1	*cV*

#### 2.2.2. Viral release by bursting

The model in (2.3) can be considered as a Markov process in which each infected cell is independent, with its own number of intracellular virions, which increases at a constant rate until the cell dies. If the cell reaches stage *n*_*I*_ and the death of the cell is virus-induced (with rate *δ*_*I*_), then the intracellular virions at the time of death will be released in a burst into the extracellular environment. On the other hand, if the infected cell is killed by the immune system (with rate *ν*_*E*_ during the eclipse phase, or rate *ν*_*I*_ during the infectious phase), it is assumed that no infectious virions will be released [11]. This can represent granzymes delivered into the infected cell which can damage the intracellular virions [23,24].

### 2.3. Average burst size and reproduction number

#### 2.3.1. Viral release by budding

Here we compute the mean burst size and reproduction number for the stochastic model of viral release by budding. The burst size is defined as the total number of virions released by an infected cell during its lifetime. We note that a cell becomes infected at the moment of viral entry, so that a cell in either the eclipse or infectious phase will be an *infected* cell. There is no viral release during the eclipse phase and the rate of viral release during the infectious phase is *p*. Thus, the mean burst size is given by

$$0\times E[t_{E}]+pE[t_{I}]=pE[t_{I}],$$

where *t*_*E*_ and *t*_*I*_ are the random variables for the length of time that an infected cell spends in the eclipse and infectious phases, respectively (i.e. *t*_*I*_ is the infectious period). When *ν*_*E*_ = *ν*_*I*_ = 0, we have $E[t_{I}]=\tau_{I}$.

We will now consider the case when the killing of infected cells due to the immune response is included (*ν*_*E*_ > 0, *ν*_*I*_ > 0). In this case, it is possible for cells to be killed by the immune system, either in the eclipse phase (before becoming infectious), or in the infectious phase before passing through all *n*_*I*_ stages. If a cell is killed during the eclipse phase, then it will never become infectious (i.e. *t*_*I*_ = 0) and therefore the burst size will be zero. Let *K* be the random variable representing the number of infectious stages that an infected cell passes through before it dies; that is, the state space of *K* is *k* ∈ {0, 1, …, *n*_*I*_}, where *k* = 0 implies the cell dies in the eclipse phase, and *k* ∈ {1, …, *n*_*I*_} implies the cell dies in the infectious stage *k*. At each infectious stage, the rate of death is *ν*_*I*_, and the rate of progression to the next stage is *δ*_*I*_ = *n*_*I*_/*τ*_*I*_. Thus, the time taken to exit each stage will be the minimum of two competing exponential random variables with rates *ν*_*I*_ and *δ*_*I*_, respectively. This minimum is an exponential random variable with rate *δ*_*I*_ + *ν*_*I*_, and is independent of whether the cell is killed by the immune response or progresses to the next stage. That is, the time spent in a given infectious stage will always be exponentially distributed with rate *δ*_*I*_ + *ν*_*I*_, even if we condition on progression to the next stage. Thus, the infectious period, *t*_*I*_, is equivalent to the sum of *K* exponential random variables, each with rate *δ*_*I*_ + *ν*_*I*_, where *K* is itself a random variable. Therefore, if *K* ∈ {1, …, *n*_*I*_}, *t*_*I*_ follows an Erlang (*K*, *δ*_*I*_ + *ν*_*I*_) distribution. If *K* = 0, then we have *t*_*I*_ = 0. The overall distribution of *t*_*I*_ will be a weighted sum of these distributions, with the weights given by the distribution of *K*, so that the infectious period, *t*_*I*_, has the following probability density:

$$\frac{d}{dt}P(t_{I}\leq t)=\sum_{k=1}^{n_{I}}\;P(K=k)\;\frac{{\left(\delta_{I}+\nu_{I}\right)}^{k}t^{k-1}\;e^{-(\delta_{I}+\nu_{I})t}}{(k-1)!}.$$

The probabilities P(K=k) giving the distribution of *K* can be found as follows. For a cell in any given eclipse stage, the exit rate of the cell out of this stage is *δ*_*E*_ + *ν*_*E*_, and when the cell exits this stage it will either die with probability *ν*_*E*_/(*δ*_*E*_ + *ν*_*E*_), or move to the next stage with probability *δ*_*E*_/(*δ*_*E*_ + *ν*_*E*_). Hence the probability that the cell will survive all *n*_*E*_ eclipse stages and progress to the infectious phase is $(\delta_{E}/{\left(\delta_{E}+\nu_{E})\right)}^{n_{E}}$. Similarly, for a cell in infectious stage *i* ∈ {1, …, *n*_*I*_ − 1}, the exit rate of the cell out of this stage is *δ*_*I*_ + *ν*_*I*_, and when the cell exits this stage it will either die with probability *ν*_*I*_/(*δ*_*I*_ + *ν*_*I*_), or move to the next infectious stage with probability *δ*_*I*_/(*δ*_*I*_ + *ν*_*I*_). Note that for the final infectious stage, *n*_*I*_, the cell will always die when exiting this stage. Therefore, the probability that an infected cell passes through *k* infectious stages before it dies is given by

2.4 $$P(K=k)=\left\{ \begin{array}{cc} 1-r_{E}^{n_{E}},\; & k=0,\;\\ r_{E}^{n_{E}}r_{I}^{k-1}(1-r_{I}),\; & k=1,\ldots ,n_{I}-1,\;\\ r_{E}^{n_{E}}r_{I}^{n_{I}-1},\; & k=n_{I},\; \end{array}\right.$$

where *r*_*E*_ = *δ*_*E*_/(*δ*_*E*_ + *ν*_*E*_), *r*_*I*_ = *δ*_*I*_/(*δ*_*I*_ + *ν*_*I*_). We note that when *ν*_*E*_ = *ν*_*I*_ = 0, one has *r*_*E*_ = *r*_*I*_ = 1, so that *K* = *n*_*I*_ with probability one. The mean infectious period can then be calculated as

$$\begin{array}{rl} E[t_{I}] & =\sum_{k=0}^{n_{I}}\;P(K=k)\;\frac{k}{\delta_{I}+\nu_{I}}\\ & =\frac{r_{E}^{n_{E}}}{\nu_{I}}\left({\left(1-r_{I}\right)}^{2}\sum_{k=1}^{n_{I}-1}[kr_{I}^{k-1}]+n_{I}r^{n_{I}-1}(1-r_{I})\right)\\ & =\frac{1}{\nu_{I}}r_{E}^{n_{E}}(1-r_{I}^{n_{I}}),\;\mathrm{for}\;\nu_{I}>0. \end{array}$$

To obtain the mean reproduction number, we multiply the mean burst size, $pE[t_{I}]$, by the mean fraction of these virions that will go on to infect new target cells,

2.5 $$\bar{R}=pE[t_{I}]\frac{\beta T_{0}}{c+\beta T_{0}}=r_{E}^{n_{E}}(1-r_{I}^{n_{I}})\frac{\;p\beta T_{0}}{\nu_{I}(c+\beta T_{0})}.$$

In the limit *ν*_*E*_ → 0 and *ν*_*I*_ → 0, we have $E[t_{I}]\to \tau_{I}$ and we regain (2.2).

#### 2.3.2. Viral release by bursting

For the model of viral release by bursting, the rate of intracellular virion production during the infectious phase is *p*, but virions will only be released in a burst if the cell is not killed by the immune system in any of the eclipse or infectious stages. The probability for a given infected cell to burst is $r_{E}^{n_{E}}r_{I}^{n_{I}}$, and given that the cell bursts, its mean infectious period will be *n*_*I*_/(*δ*_*I*_ + *ν*_*I*_). Thus, the mean burst size is

$$r_{E}^{n_{E}}r_{I}^{n_{I}}\frac{\;pn_{I}}{\delta_{I}+\nu_{I}},$$

and the mean reproduction number is

2.6 $$\bar{R}=r_{E}^{n_{E}}r_{I}^{n_{I}+1}\frac{\;p\tau_{I}\beta T_{0}}{c+\beta T_{0}}.$$

In the limit *ν*_*E*_ → 0 and *ν*_*I*_ → 0, we have *r*_*E*_ = *r*_*I*_ = 1, and we regain (2.2).

### 2.4. Burst size probability distribution

#### 2.4.1. Viral release by budding

Let *B* be the random variable representing the viral burst size, i.e. the total number of virions released by an infected cell during its lifetime. To find the distribution of *B*, we consider the dynamics of viral release from a single infected cell. As explained in the previous section, an infected cell will progress through a (random) number of infectious stages, *K* ∈ {0, 1, …, *n*_*I*_}, before it dies. The probability distribution of *K* is given in (2.4). We will first consider the burst size distribution conditioned on the value of *K*, and then compute a weighted sum over the possible values of *K*. If *K* = 0, then the cell dies before entering the infectious phase and the burst size is zero. If *K* = *k* with *k* > 0, then there are *k* − 1 progression events (from one infectious stage to the next) and *b* virion release events before the cell dies in the *k*th infectious stage. During the infectious phase, a cell is assumed to release virions at a constant rate, *p*, with an exponentially distributed time between the release of each new virion. Hence, each virion release event has probability *p*/(*p* + *δ*_*I*_ + *ν*_*I*_). We thus conclude that the total number of virions released by the cell before it dies is a negative binomial random variable describing the number of ‘successes’ before the *k*th ‘failure’, where a ‘success’ is the release of a virion and a ‘failure’ is leaving the stage. Therefore, given that the cell progresses through exactly *K* = *k* > 0 infectious stages, the burst size *B* follows a negative binomial distribution, with shape parameter *k* and success probability *p*/(*p* + *δ*_*I*_ + *ν*_*I*_). Once we sum over the possible values of *K*, the probability mass function (p.m.f.) for the number of virions released from an infected cell is

2.7 $$\begin{array}{rl} P(B=b) & =\sum_{k=0}^{n_{I}}\;P(B=b|K=k)\;P(K=k)\\ & =(1-r_{E}^{n_{E}})\delta_{b,0}+r_{E}^{n_{E}}(\sum_{k=1}^{n_{I}-1}\;f\;(b,k)\frac{\delta_{I}^{k-1}\nu_{I}}{{\left(p+\delta_{I}+\nu_{I}\right)}^{k}}\\ & \;+\;f\;(b,n_{I})\frac{\delta_{I}^{n_{I}-1}(\delta_{I}+\nu_{I})}{{\left(p+\delta_{I}+\nu_{I}\right)}^{n_{I}}}), \end{array}$$

for *b* ∈ {0, 1, 2, …}, where *δ*_*b*,0_ represents the Kronecker delta, and

2.8 $$f\;(b,k)=\frac{(k+b-1)!}{(k-1)!b!}{\left(\frac{\;p}{p+\delta_{I}+\nu_{I}}\right)}^{b}.$$

If *ν*_*E*_ = *ν*_*I*_ = 0, the burst size distribution becomes a negative binomial with shape parameter *n*_*I*_ and success probability *p*/(*p* + *δ*_*I*_), so that we can write

2.9 $$P(B=b)=\left(\begin{array}{c} n_{I}+b-1\\ b \end{array}\right){\left(\frac{\;p}{p+\delta_{I}}\right)}^{b}{\left(\frac{\delta_{I}}{p+\delta_{I}}\right)}^{n_{I}},$$

for *b* ∈ {0, 1, 2, …}. The expectation and variance of this negative binomial distribution are given by

$$E[B]=\frac{\;pn_{I}}{\delta_{I}}=p\tau_{I},$$

and

$$\mathrm{Var}[B]=E[B]\left(1+\frac{E[B]}{n_{I}}\right)=\frac{\;pn_{I}}{\delta_{I}}+\frac{\;p^{2}n_{I}}{\delta_{I}^{2}}.$$

We note that if the infectious period is assumed to be exponentially distributed (*n*_*I*_ = 1), then the distribution in (2.9) becomes geometric.

#### 2.4.2. Viral release by bursting

When *ν*_*I*_ = 0, the probability distribution of the burst size is equal for the two models of budding and bursting, since the number of virions produced by an infected cell has the same distribution in both instances, but they are either released from the cell gradually in the case of budding, or remain in the cell until it dies in the case of bursting. However, when infectious cells can be killed by the immune system, the two strategies produce different burst size distributions. In the budding strategy, some virions may be released from an infectious cell before the cell is killed by the immune system, but in the bursting strategy, if an infectious cell is killed by the immune system before it bursts, then all intracellular virions are assumed to be eliminated in the process and the corresponding burst size is zero. Hence, in the case of bursting, virions will only be released from the infected cell if the cell is not killed by the immune system, and it exits the final infectious stage by bursting. Given that a burst does occur, the burst size will be negative binomially distributed with shape parameter *n*_*I*_ and success probability *p*/(*p* + *δ*_*I*_ + *ν*_*I*_). Thus, the burst size probability distribution is given by

2.10 $$\begin{array}{rl} P(B=b) & =(1-r_{E}^{n_{E}}r_{I}^{n_{I}})\delta_{b,0}\\ & \;+r_{E}^{n_{E}}f\;(b,n_{I}){\left(\frac{\delta_{I}}{p+\delta_{I}+\nu_{I}}\right)}^{n_{I}}, \end{array}$$

for *b* ∈ {0, 1, 2, …}, where *δ*_*b*,0_ represents the Kronecker delta, and *f*(*b*, *k*) is defined in (2.8).

The burst size p.m.f.s and means for the budding and bursting models are summarized in table 2.

**Table 2.** Summary of the burst size and reproduction number distributions and means for the different model assumptions of viral release by budding and bursting, defined for *b*, *r* ∈ {0, 1, 2, …}. *δ*_*b*,0_ represents the Kronecker delta and we define *r*_*E*_ = *δ*_*E*_/(*δ*_*E*_ + *ν*_*E*_), *r*_*I*_ = *δ*_*I*_/(*δ*_*I*_ + *ν*_*I*_). *f*(*b*, *k*) is defined in (2.8). Note that these reproduction number distributions were calculated for the case of a constant number of uninfected target cells (i.e. Case 1), where each virion has probability *θ* to infect a new cell. For Case 2, in which the depletion of uninfected target cells is considered, the approach in (2.12) should be followed, using the recursively defined probabilities, *p*_*r*_[*b*].

burst size distribution	
budding	$P(B=b)=(1-r_{E}^{n_{E}})\delta_{b,0}+r_{E}^{n_{E}}\left(\sum_{k=1}^{n_{I}-1}\;f(b,k)\frac{\delta_{I}^{k-1}\nu_{I}}{{\left(p+\delta_{I}+\nu_{I}\right)}^{k}}+f(b,n_{I})\frac{\delta_{I}^{n_{I}-1}(\delta_{I}+\nu_{I})}{{\left(p+\delta_{I}+\nu_{I}\right)}^{n_{I}}}\right)$
bursting	$P(B=b)=(1-r_{E}^{n_{E}}r_{I}^{n_{I}})\delta_{b,0}+r_{E}^{n_{E}}f(b,n_{I}){\left(\frac{\delta_{I}}{p+\delta_{I}+\nu_{I}}\right)}^{n_{I}}$

mean burst size	
budding	$\bar{B}=r_{E}^{n_{E}}(1-r_{I}^{n_{I}})\frac{\;p}{\nu_{I}}$
bursting	$\bar{B}=r_{E}^{n_{E}}r_{I}^{n_{I}}\frac{\;pn_{I}}{\delta_{I}+\nu_{I}}$

reproduction number distribution	
budding	$P(R=r)=(1-r_{E}^{n_{E}})\delta_{r,0}+r_{E}^{n_{E}}\left(\sum_{k=1}^{n_{I}-1}\;\left(\begin{array}{c} k+r-1\\ r \end{array}\right)\frac{{\left(\theta p\right)}^{r}\delta_{I}^{k-1}\nu_{I}}{{\left(\theta p+\delta_{I}+\nu_{I}\right)}^{r+k}}+\left(\begin{array}{c} n_{I}+r-1\\ r \end{array}\right)\frac{{\left(\theta p\right)}^{r}\delta_{I}^{n_{I}-1}(\delta_{I}+\nu_{I})}{{\left(\theta p+\delta_{I}+\nu_{I}\right)}^{r+n_{I}}}\right)$
bursting	$P(R=r)=(1-r_{E}^{n_{E}}r_{I}^{n_{I}})\delta_{r,0}+r_{E}^{n_{E}}\left(\begin{array}{c} n_{I}+r-1\\ r \end{array}\right)\frac{{\left(\theta p\right)}^{r}\delta_{I}^{n_{I}}}{{\left(\theta p+\delta_{I}+\nu_{I}\right)}^{r+n_{I}}}$

mean reproduction number	
budding	$\bar{R}=r_{E}^{n_{E}}(1-r_{I}^{n_{I}})\frac{\;p\theta }{\nu_{I}}$
bursting	$\bar{R}=r_{E}^{n_{E}}r_{I}^{n_{I}+1}p\tau_{I}\theta$

### 2.5. Reproduction number probability distribution

We now return to *R*, the random variable representing the cell-level reproduction number; that is, the number of secondary infections produced by a single infected cell in an otherwise susceptible cell population. Our aim in this section is to find the p.m.f. of *R*, which may be written in terms of the burst size probabilities as

$$P(R=r)=\sum_{b=0}^{+\infty }\;P(R=r|B=b)\;P(B=b),$$

with *r* a non-negative integer. Hence, we will find the probabilities P(R=r∣B=b) (number of secondary infections produced, given that the initial infected cell releases *B* = *b* virions during its lifetime), so that together with the probability distribution of *B* ((2.7) for budding and (2.10) for bursting), we can compute the probability distribution of *R*.

The only possible outcomes for a released virion are (i) that it is lost or (ii) that it infects a target cell. Free virions are cleared with rate *c*, and infect new cells with rate *βT*, which depends on the number of available target cells, *T*. Usually, calculations of the reproduction number assume that the number of infectious viral particles is small enough so that the number of (uninfected) target cells is not significantly altered by infection events. Thus, the number of target cells is usually assumed constant [25], as in §2.5.1. However, if the number of target cells at the start of infection is small and the infection rate, *β*, is large, it will be important to consider the transition of target cells to infected cells, since the depletion of uninfected target cells will affect the ability of extracellular viral particles (released from infected cells) to infect new cells. The number of secondary infections caused by a single infected cell will depend on: (i) competition between virions produced by the initial infected cell for available target cells and (ii) competition for target cells from virions being produced during secondary infections. Here, in §2.5.2, we will address the former source of competition, while ignoring the second; that is, we consider depletion of target cells due to secondary infections caused by viral particles released from the initially infected cell, but do not consider depletion of target cells due to viral particles released from subsequently infected cells.

#### 2.5.1. Target cell number is constant (Case 1)

If the number of uninfected target cells is sufficiently large, their loss due to infection can be neglected, and their number assumed to be constant. In this case, extracellular virions are independent of each other, in the sense that they will have the same probability of successfully infecting a target cell, independently of the fate of other such virions. For each infectious virion released from an initially infected cell, the probability that it will infect a target cell is

$$\theta =\frac{\beta T_{0}}{c+\beta T_{0}},$$

where *β* is the rate at which extracellular virions infect target cells, *T*_0_ is the constant number of target cells, and *c* is the rate of loss of extracellular virions. Therefore, the number of secondary infections will be the sum of *b* independent and identical Bernoulli random variables, and *R*|*B* = *b*, follows a binomial distribution with parameters (*b*, *θ*). We then have, for *r* a non-negative integer,

$$P(R=r)=\sum_{b=r}^{+\infty }\;\left(\begin{array}{c} b\\ r \end{array}\right)\;\theta^{r}\;{\left(1-\theta \right)}^{b-r}\;P(B=b).$$

Substituting in the p.m.f of the burst size distribution from (2.7) (budding model) or (2.10) (bursting model), we find that the p.m.f of the reproduction number has the same form as the burst size p.m.f., but with the parameter *p* replaced by *θp*, which is equivalent to assuming that secondary infections are produced at rate *θp*. This makes sense since each virion released from an infected cell is expected to infect a new cell with probability *θ*, and when considering only the number of secondary infections produced, we ignore the timescale of these events. The reproduction number p.m.f.s and means for the budding and bursting models are provided in table 2.

If *ν*_*E*_ = *ν*_*I*_ = 0, since the burst size follows a negative binomial distribution (see (2.9)), then the number of secondary infections also follows a negative binomial distribution, with shape parameter *n*_*I*_ and success probability *θp*/(*θp* + *δ*_*I*_). The p.m.f. of the reproduction number is

2.11 $$P(R=r)=\left(\begin{array}{c} n_{I}+r-1\\ r \end{array}\right){\left(\frac{\theta p}{\theta p+\delta_{I}}\right)}^{r}{\left(\frac{\delta_{I}}{\theta p+\delta_{I}}\right)}^{n_{I}}.$$

#### 2.5.2. Target cell number is limiting (Case 2)

The probability of a given virion successfully infecting a cell before it is lost depends on the number of available target cells. Therefore, if the number of target cells is small, it is essential to take into account their reduction as they become infected. In this case, the virions cannot be treated as independent, but their probability of infecting a cell will depend on how many target cells are still available after previous infection events.

Let us assume that there are *T*_0_ target cells in the population to begin with, and *B* = *b* infectious virions have been released by one infected cell during its infectious phase. Since we are only interested in the number of secondary infections generated by a single infected cell, and not the times of those infection events, one can assume that at discrete time steps, a virion is chosen uniformly at random out of the *B* = *b* virions, to either be lost or infect a target cell. In this way, we can then set up a Markov chain to calculate the probability distribution of the number of secondary infections generated by *B* = *b* extracellular virions produced by a single infected cell.

**Figure 2.**
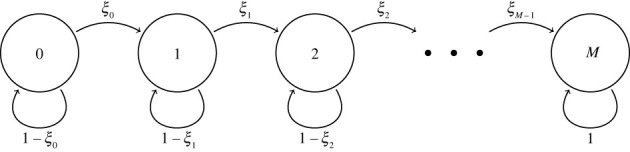
Counting secondary infections using a Markov chain. At each of *b* steps, one for each released virion, the process either moves one state to the right, corresponding to infection of a target cell, or stays put, corresponding to loss of a virion. *ξ*_*i*_ = *β*(*T*_0_ − *i*)/(*β*(*T*_0_ − *i*) + *c*) is the probability of moving from state *i* to *i* + 1, where *T*_0_ is the initial number of uninfected target cells available. The position after *b* steps is the number of secondary infections generated by *b* extracellular virions.

Let us define the discrete-time Markov chain, Y= $\left\{Y_{n}\;:\;n\in \{0,1,2,\ldots ,b\}\right\}$, where *Y*_*n*_ denotes the number of secondary infections that have occurred after *n* of the infectious extracellular virions have either been lost or infected an available target cell. The initial state of the process will be *Y*_0_ = 0 and the state space of Y will be given by $S_{Y}=\{0,1,2,\ldots ,M\}$, where *M* = min{*b*, *T*_0_} is the maximum number of secondary infections that can take place. The Markov chain Y, shown in figure 2, can be defined by the following one-step transition probabilities, for states $i,j\in S_{Y}$. For *i* < *M*,

$$p_{i,j}=P(Y_{n+1}=j|Y_{n}=i)=\left\{ \begin{array}{cc} \xi_{i},\; & \mathrm{if}\;j=i+1,\;\\ 1-\xi_{i},\; & \mathrm{if}\;j=i,\;\\ 0,\; & \mathrm{otherwise},\; \end{array}\right.$$

where *ξ*_*i*_ = *β*(*T*_0_ − *i*)/(*β*(*T*_0_ − *i*) + *c*). If the number of secondary infections reaches *M*, we have

$$p_{M,j}=\left\{ \begin{array}{cc} 1,\; & \mathrm{if}\;j=M,\;\\ 0,\; & \mathrm{otherwise}.\; \end{array}\right.$$

The probability that *r* secondary infections will occur (with *r* a non-negative integer), given that the cell released *b* infectious virions, is given by $P(R=r|B=b)=P(Y_{b}=r|Y_{0}=0)$, which is the probability that the process Y is in state *r* after the *b* infectious extracellular virions have either been lost or infected target cells. For ease of notation, we write $p_{r}[b]=P(Y_{b}=r|Y_{0}=0)$. Making use of first-step arguments, these probabilities can be calculated recursively, as follows. We have *p*_0_[0] = 1. Then for *b* ≥ 1,

$$p_{r}[b]=\left\{ \begin{array}{cc} p_{0}[b-1]p_{0,0},\; & \mathrm{if}\;r=0,\;\\ p_{r-1}[b-1]p_{r-1,r}+p_{r}[b-1]p_{r,r},\; & \mathrm{if}\;0<r\leq M.\; \end{array}\right.$$

Thus, substituting the one-step transition probabilities from above, we have

$$p_{r}[b]=\left\{ \begin{array}{cc} {\left(1-\xi_{0}\right)}^{b},\; & \mathrm{if}\;r=0,\;\\ p_{r-1}[b-1]\xi_{r-1}+p_{r}[b-1](1-\xi_{r}),\; & \mathrm{if}\;0<r\leq \min(b,T_{0}),\;\\ 0,\; & \mathrm{if}\;r>\min(b,T_{0}).\; \end{array}\right.$$

We can solve the previous system sequentially to obtain *p*_*r*_[*b*] for all required values of *r* and *b*. We get

2.12 $$P(R=r)=\sum_{b=0}^{+\infty }\;p_{r}[b]\;P(B=b).$$

If the initial number of target cells, *T*_0_, is large enough so that only a small fraction is going to become infected by the virions produced by a single infected cell, then it is reasonable to approximate *ξ*_*i*_ = *θ* for all *i*. This approximation leads to Case 1, where

$$p_{r}[b]=\left(\begin{array}{c} b\\ r \end{array}\right)\;\theta {\;}^{r}\;{\left(1-\theta \right)}^{b-r},\;0\leq r\leq b.$$

### 2.6. Probability of viral extinction

It is useful to also calculate the probability of viral extinction during the early stages of infection. When the number of target cells is assumed constant, the initial dynamics of the models considered can be described by branching processes, in which each infected cell produces a number of new infected cells according to the reproduction number distribution. In such a branching process, the probability of extinction starting with one infected cell is equal to the smallest fixed point of the probability generating function (p.g.f.) of the offspring distribution (see ch. 4 of Allen [26]). If the branching process is counting the number of infected cells at each generation, then the offspring distribution will be the reproduction number distribution.

In the case of budding, the p.g.f. of the reproduction number (when the number of target cells is constant) is given by

2.13 $$\begin{array}{rl} \pi_{\mathrm{bud}}(s) & =1-r_{E}^{n_{E}}[1-\sum_{k=1}^{n_{I}}\frac{\tau_{I}\nu_{I}n_{I}^{k-1}}{{\left(n_{I}+\tau_{I}\nu_{I}+(1-s)\tau_{I}\theta p\right)}^{k}}\\ & \;-{\left(\frac{n_{I}}{n_{I}+\tau_{I}\nu_{I}+(1-s)\tau_{I}\theta p}\right)}^{n_{I}}]. \end{array}$$

Further details to show how this p.g.f. was obtained can be found in Williams [27, p. 170].

In the case of bursting, the p.g.f. of the reproduction number (when the number of target cells is constant) is given by

2.14 $$\begin{array}{r} \pi_{\mathrm{burst}}(s)=1-r_{E}^{n_{E}}r_{I}^{n_{I}}\left[1-{\left(\frac{n_{I}+\tau_{I}\nu_{I}}{n_{I}+\tau_{I}\nu_{I}+(1-s)\tau_{I}\theta p}\right)}^{n_{I}}\right]. \end{array}$$

We note that when *ν*_*I*_ = 0, *π*_bud_ and *π*_burst_ are identical, since the reproduction number distribution is the same for the budding and bursting cases.

When *n*_*I*_ = 1, corresponding to an exponentially distributed infectious period, the fixed points of the functions *π*_bud_ and *π*_burst_ can be found explicitly. For budding and *n*_*I*_ = 1, the solutions of *π*_bud_(*s*) = *s* are $s_{1}^{\mathrm{bud}}=1$ and

$$s_{2}^{\mathrm{bud}}=1-r_{E}^{n_{E}}\left(1-\frac{1}{{\bar{R}}_{\mathrm{bud}}}\right),$$

where ${\bar{R}}_{\mathrm{bud}}=r_{E}^{n_{E}}(\theta p/\delta_{I}+\nu_{I})$ is the mean reproduction number in the budding case (*n*_*I*_ = 1). Similarly, for bursting and *n*_*I*_ = 1, the solutions to *π*_burst_(*s*) = *s* are $s_{1}^{\mathrm{burst}}=1$ and

$$s_{2}^{\mathrm{burst}}=1-r_{E}^{n_{E}}r_{I}\left(1-\frac{1}{{\bar{R}}_{\mathrm{burst}}}\right),$$

where ${\bar{R}}_{\mathrm{burst}}=r_{E}^{n_{E}}r_{I}(\theta p/\delta_{I}+\nu_{I})=r_{I}{\bar{R}}_{\mathrm{bud}}$. The smallest fixed point of the p.g.f. gives the probability of viral extinction starting with one infected cell, so that in each case extinction will be certain if *s*_2_ ≥ 1 (corresponding to $\bar{R}\leq 1$) and will occur with probability *s*_2_ if *s*_2_ < 1 (corresponding to $\bar{R}>1$). Since ${\bar{R}}_{\mathrm{burst}}\leq {\bar{R}}_{\mathrm{bud}}$ and $s_{2}^{\mathrm{burst}}\geq s_{2}^{\mathrm{bud}}$, extinction will always be equally or more likely to occur in the bursting case than the budding one. In particular, when $1<{\bar{R}}_{\mathrm{bud}}\leq 1/r_{I}$, extinction is certain in the bursting case, but not in the budding one. For *n*_*I*_ > 1, the smallest fixed points of the p.g.f.s can be found numerically, giving the probability of viral extinction starting with one infected cell. Since each initial infected cell is independent, the probability of extinction starting with one infected cell can easily be used to find the probability of extinction starting with multiple infected cells [26].

## 3. Numerical results

In this section, we present the sensitivity of the reproduction number distribution and probability of viral extinction computed in §2 to different models of viral release and parameter values. We ignore any immune killing of eclipse-phase cells, so that *ν*_*E*_ = 0 and every infected cell is assumed to survive the eclipse phase and enter the infectious phase.

### 3.1. Infection rate

We first consider the model without immune killing of infectious cells, corresponding to *ν*_*I*_ = 0. In this case, the probability distribution of the number of virions released by an infected cell during its lifetime is the same for either model of viral release (budding or bursting). For both models, the burst size follows a negative binomial distribution with mean *pτ*_*I*_ and shape parameter *n*_*I*_ (see (2.9)). Once virions are released from an infected cell, the rate at which they infect new cells depends on *β* and on the number of target cells available to be infected, *T*. Section 2.5 described two cases to consider when calculating the distribution of the reproduction number, *R*. When the number of target cells is not limiting, since the population is very large or is constantly replenished (Case 1), the reproduction number follows the negative binomial distribution in (2.11) (for *ν*_*E*_ = *ν*_*I*_ = 0). The shape parameter of this distribution is *n*_*I*_, and the mean number of secondary infections produced is $\bar{R}=\theta p\tau_{I}$, where *θ* = *βT*_0_/(*c* + *βT*_0_) is the probability for a given released virion to infect a target cell. Alternatively, if the number of target cells is limiting (Case 2), the reproduction number distribution can be calculated using (2.12), in which the depletion of target cells due to infection is taken into account.

When the value of $\bar{R}$ is much smaller than *T*_0_, and only a small fraction of the target cells are likely to become infected, the probability distributions of *R* for Case 1 and Case 2 are similar. The smaller the difference between the number of target cells and the expected number to become infected (i.e. $T_{0}-\bar{R}$), the greater the difference of the distributions obtained from the two methods become.

**Figure 3.**
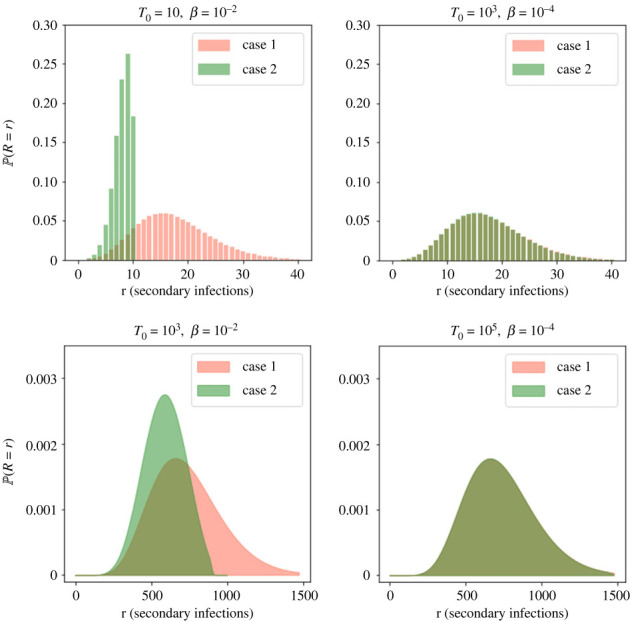
Histograms for the probability distribution of the reproduction number, *R*, for different values of *β* and *T*_0_. For each pair of parameter values, the distributions obtained from methods of Case 1 using (2.11) (number of target cells is constant) and Case 2 using (2.12) (number of target cells decreases as they become infected) are shown. All other parameter values are fixed to the values in table 3, with *ν*_*I*_ = 0 (so the reproduction number distribution is equivalent under budding or bursting assumptions).

Figure 3 shows the different probability distributions of *R* calculated with Case 1 and Case 2 methods, for some key values of the infection rate, *β*, and number of target cells, *T*_0_. All other parameters are fixed to the values in table 3, with *ν*_*I*_ = 0. The Case 1 method assumes that there is a constant population of *T*_0_ target cells which does not decrease as cells become infected. On the other hand, Case 2 assumes that there is a population of *T*_0_ target cells to begin with, but each secondary infection reduces this number by one, so that it is not possible for more than *T*_0_ secondary infections to occur. The two plots in the top row of figure 3 have parameter values of *β* and *T*_0_, such that *θ* = 0.014 and the mean reproduction number is $\bar{R}=17.6$ (for Case 1, from (2.2)). However for the plot on the top left, $T_{0}=10<\bar{R}$, making the distribution of *R* very different, depending on whether it is calculated using the method for Case 1 or Case 2. When $T_{0}=10^{3}\gg \bar{R}$, the two distributions become very similar. For the plots on the bottom row of figure 3, the values of *β* and *T*_0_ give *θ* = 0.59 and $\bar{R}=735$. For the plot on the bottom left, where *T*_0_ = 10^3^ is only slightly larger than $\bar{R}$, there is a clear difference between the two distributions. When *T*_0_ is increased to 10^5^ in the plot on the bottom right, this difference becomes much smaller.

**Table 3.** Model parameters with their descriptions and units. We define *δ*_*E*_ = *n*_*E*_/*τ*_*E*_, *δ*_*I*_ = *n*_*I*_/*τ*_*I*_, and *θ* = *βT*_0_/(*c* + *βT*_0_). Values are given for some parameters, which have been used to obtain numerical results in §3. In all numerical results, we set *ν*_*E*_ = 0, which means that the values of *τ*_*E*_ and *n*_*E*_ (determining the distribution of the eclipse phase) do not affect the burst size or reproduction number distribution.

parameter	description	units	value
*p*	viral release rate (budding) or intracellular production rate (bursting)	virions · (cell · day)^−1^	1000
*τ*_*E*_	mean of Erlang-distributed eclipse phase in the absence of immune killing	days	n.a.
*n*_*E*_	number of stages of the Erlang-distributed eclipse phase	—	n.a.
*ν*_*E*_	immune clearance rate of eclipse-phase cells	(cell · day)^−1^	0
*τ*_*I*_	mean of Erlang-distributed infectious period in the absence of immune killing	days	1.25
*n*_*I*_	number of stages of the Erlang-distributed infectious period	—	10
*ν*_*I*_	immune clearance rate of infectious cells	(cell · day)^−1^	varied
*β*	infection rate	(cell · virion · day)^−1^	10^−4^
*c*	rate of loss of extracellular virions	(virion · day)^−1^	7
*T*_0_	initial number of target cells	cells	10^5^

**Figure 4.**
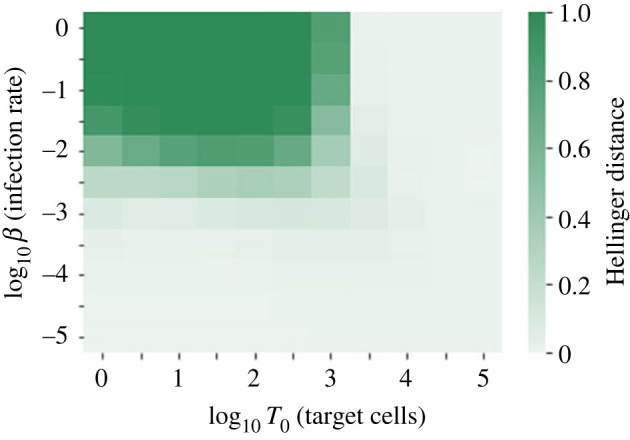
Heatmap showing the Hellinger distance between the two distributions of the reproduction number, *R*, calculated using Case 1 (2.11) and Case 2 (2.12), for different values of *β* and *T*_0_. All other parameter values are fixed to the values in table 3, with *ν*_*I*_ = 0 (so the reproduction number distribution is equivalent under budding or bursting assumptions).

The heatmap in figure 4 illustrates the difference between the two distributions of Case 1 and Case 2 across a wider range of values of *β* and *T*_0_. The difference between the distributions is quantified by the Hellinger distance, defined by

$$H(P,Q)=\frac{1}{\sqrt{2}}\sqrt{\sum_{i}{\left(\sqrt{\;p_{i}}-\sqrt{q_{i}}\right)}^{2}},$$

for two discrete probability distributions, *P* = {*p*_0_, *p*_1_, *p*_2_, …} and *Q* = {*q*_0_, *q*_1_, *q*_2_, …}. *H*(*P*, *Q*) gives a value between 0 and 1, where 0 indicates that the two distributions are identical. In general, for larger values of the infection rate, *β*, and smaller values of *T*_0_, the distributions become more different, since these parameter values will mean that the virus is likely to infect a greater fraction of the available target cells.

For the value of *β* = 10^−4^ (cell · virion · day)^−1^ in table 3, the mean reproduction number will be much smaller than *T*_0_, for any $T_{0}\in N$. Therefore the distribution of *R* will be very similar using either method, and we will assume a constant number of target cells (Case 1) to calculate the reproduction number distributions in the following sections.

### 3.2. Immune killing

**Figure 5.**
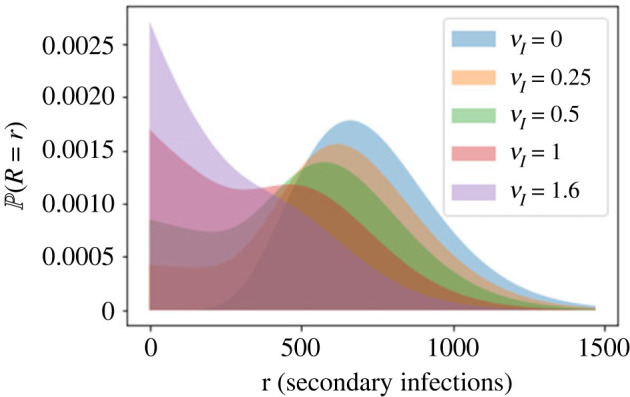
Reproduction number probability distributions calculated using the expression in table 2 for the model of viral release by budding, for different values of the immune killing rate of infectious cells, *ν*_*I*_. Other parameters are set to the values in table 3. The values of *ν*_*I*_ used are 0, 0.25, 0.5, 1 and 1.6, per cell per day, corresponding to values of $\bar{R}$ of 735, 623, 535, 407 and 308, respectively.

**Figure 6.**
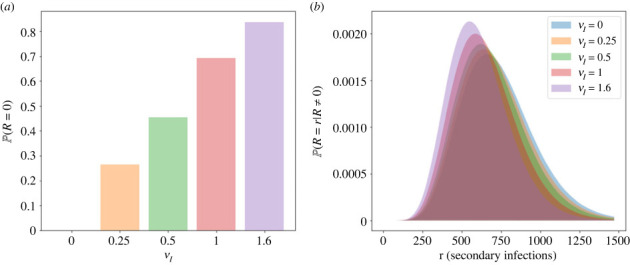
Reproduction number probability distributions calculated using the expression in table 2 for the model of viral release by bursting, for different values of the immune killing rate of infectious cells, *ν*_*I*_. Other parameters are set to the values in table 3. The values of *ν*_*I*_ used are 0, 0.25, 0.5, 1 and 1.6, per cell per day, corresponding to values of $\bar{R}$ of 735, 524, 377, 201 and 99, respectively. (*a*) Probability of zero secondary infections for each value of *ν*_*I*_. (*b*) Probability distribution of *R* conditioned on positive values of the reproduction number.

*In vitro* experiments have previously been used to obtain estimates for viral dynamics parameters. For example, Liao *et al.* [2] fitted a mathematical model similar to (2.1) with *ν*_*E*_ = *ν*_*I*_ = 0 to data of *in vitro* Ebola virus infection. In another recent study, Yan *et al.* [7] fitted a similar model to *in vitro* data and compared estimates of growth rate, reproduction number and generation time, for six influenza A strains. Studies like these are very useful for determining many key model parameters; however, they do not allow parameters that represent *in vivo* processes, such as immune system clearance of infected cells, to be estimated. Here we show that the reproduction number distribution is very sensitive to the rate at which the immune system is assumed to clear infected cells *in vivo*. Figures 5 and 6 illustrate the reproduction number distribution for different rates of immune killing of infectious cells, for the models of viral release by budding and bursting, respectively. In the model of budding, infectious cells are assumed to produce new infections at a constant rate, *θp*, until they die. An increased rate of immune clearance means that the infectious period distribution is changed, with more cells dying earlier. Therefore, it becomes more likely for low numbers of secondary infections to be produced.

In the bursting model, the reproduction number probability distribution is given by a zero-inflated negative binomial distribution; if an infected cell bursts and releases virions, then the number of secondary infections caused by the cell follows a negative binomial distribution with shape parameter *n*_*I*_ and probability *θp*/(*θp* + *δ*_*I*_ + *ν*_*I*_). The probability of zero secondary infections is inflated, since if the initial infected cell is killed by the immune system before it bursts, then it is assumed that zero virions will be released and zero secondary infections can be produced. Thus, for larger rates of immune clearance, the probability of zero secondary infections increases. Given that a cell does burst and release virions, the distribution of time until the cell bursts is also affected by the rate of immune clearance. The reason for this is that the immune response introduces a competition between the two mechanisms of death for infected cells. This has the effect of reducing the mean time to cell burst, since cells that would have taken longer to burst are more likely to be cleared by the immune response before they do so. Hence the mean number of virions released from bursting cells and the mean number of secondary infections is also reduced.

**Figure 7.**
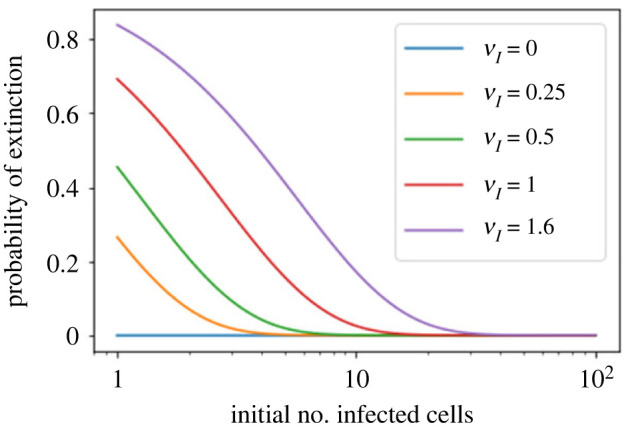
Probability of viral extinction as a function of the initial number of infected cells, for the model of viral release by bursting, for different values of the immune killing rate of infectious cells, *ν*_*I*_. The probability of extinction starting with one cell is calculated by numerically finding the smallest fixed point of the p.g.f. in (2.14). To obtain the probability of extinction starting with *i* infected cells, one takes the probability for one cell to the power of *i*. Other parameters are set to the values in table 3.

These results for the reproduction number distribution can be translated into the probability of viral extinction, using the p.g.f.s presented in §2.6. Figure 7 shows the effect of immune killing on the probability of viral extinction for the bursting model. When *ν*_*I*_ = 0, corresponding to an *in vitro* situation without the immune clearance of infected cells, the model predicts almost zero chance of viral extinction starting from one infected cell, for the parameters used here. As the immune clearance rate increases, viral extinction becomes much more likely. These results highlight how processes *in vivo*, such as the immune system clearance of infected cells, may alter the expected probability of extinction obtained from models of *in vitro* experiments. There are also many other *in vivo* biological mechanisms to consider, other than the rate at which the immune system clears infected cells, which are not studied here but may also impact the reproduction number distribution. Some of these are discussed in §4.

### 3.3. Number of infectious stages

We now study the impact of the number of infectious phase stages, given by the parameter *n*_*I*_, on the reproduction number distribution and probability of viral extinction for the model with bursting. We note that the number of infectious stages, *n*_*I*_, is varied, but with the mean of the Erlang-distributed time to cell burst, *τ*_*I*_, kept constant. Thus, as *n*_*I*_ is varied, *δ*_*I*_ = *n*_*I*_/*τ*_*I*_ also changes, since it depends on *n*_*I*_. Other parameters are set to their values in table 3 and we use an immune clearance rate of *ν*_*I*_ = 1.6 per cell per day. This implies that the immune system killing of infectious cells occurs on average two times faster than virus-induced cell death, which is assumed to take *τ*_*I*_ = 1.25 days on average.

**Figure 8.**
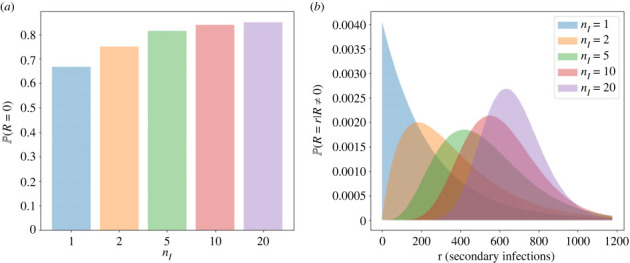
Reproduction number distributions calculated using the expression in table 2 for the bursting model, for different numbers of infectious phase stages, *n*_*I*_. For each value of *n*_*I*_, the mean of the Erlang-distributed time until cell burst is kept fixed to the value of *τ*_*I*_ in table 3. Other parameters are also set to the values in table 3, and the rate of immune killing of infectious cells is *ν*_*I*_ = 1.6 per cell per day. (*a*) Probability of zero secondary infections for each value of *n*_*I*_. (*b*) Probability distribution of *R* conditioned on positive values of the reproduction number.

Figure 8 shows the bursting model reproduction number distribution for different values of *n*_*I*_. As the number of infectious stages increases, the variance of the Erlang burst time distribution decreases and the distribution becomes more tightly centred around the mean. On the other hand, the exponential killing time distribution is not changed. Since the exponential distribution allows times very close to zero, the killing time is likely to be smaller than the burst time, leading to a higher chance that killing by the immune system will occur first. Therefore, there is a higher probability of zero secondary infections for larger values of *n*_*I*_ (figure 8*a*). However, if the infected cell does burst, it will release a larger number of virions on average when the value of *n*_*I*_ is higher. Therefore, when conditioned on positive numbers of secondary infections, the reproduction number distribution moves to the right with increasing *n*_*I*_ (figure 8*b*). For the parameter values in table 3 and the values of *n*_*I*_ considered, the overall mean reproduction number, given in (2.6), increases with the value of *n*_*I*_.

**Figure 9.**
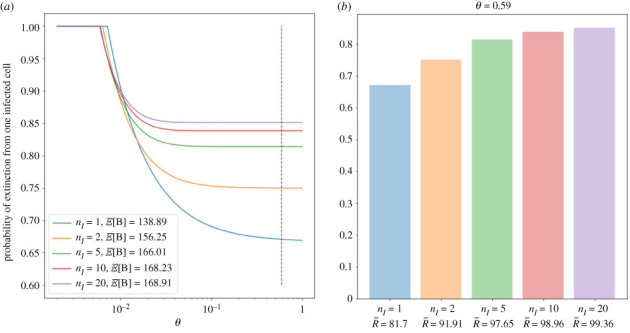
Plots to show how the probability of viral extinction depends on the number of infectious phase stages, *n*_*I*_, for the model of viral release by bursting. The probability of extinction is calculated by numerically finding the smallest fixed point of the p.g.f. in (2.14). (*a*) The probability of viral extinction starting from one infected cell as a function of *θ*, for different values of *n*_*I*_. The value of *θ* obtained from the values of *β*, *c* and *T*_0_ in table 3 is indicated by the dashed line. For each value of *n*_*I*_, the mean of the Erlang-distributed time until cell burst is kept fixed to the value of *τ*_*I*_ in table 3. The viral production rate is also fixed to the value of *p* in table 3, and the rate of immune killing of infectious cells is *ν*_*I*_ = 1.6 per cell per day. (*b*) The probability of viral extinction starting from one infected cell is shown for different values of *n*_*I*_, for *θ* = 0.59 (the value indicated by the dashed line in the left plot). Along with the number of infectious stages, the mean reproduction number increases along the *x*-axis.

Figure 9 presents some interesting results about the probability of viral extinction, which is found by numerically calculating the smallest fixed point of the p.g.f. in (2.14). Figure 9*a* shows how the probability of extinction starting from one infected cell changes as a function of *θ*, for different values of *n*_*I*_, where *θ* is the probability that a given extracellular virion goes on to infect a new cell rather than be cleared. When *θ* is very small, extinction is certain because the mean reproduction number is below 1. When *θ* grows large enough to increase the mean reproduction number above 1 (i.e. θ>1/E[B]), then the probability of extinction becomes less than 1. As seen in the legend of the plot, the mean burst size increases with *n*_*I*_. Thus, as *n*_*I*_ increases, the minimum value of *θ* needed to satisfy the condition θ>1/E[B] becomes smaller. However, once this threshold value of *θ* is exceeded, larger values of *n*_*I*_ cause the probability of extinction to decay more slowly as a function of *θ*, compared with smaller values of *n*_*I*_. As a result, we see that for small values of *θ*, there is a larger probability of extinction for smaller *n*_*I*_, but as *θ* grows, the ordering changes. Eventually, for large enough values of *θ*, the probability of extinction is an increasing function of *n*_*I*_. This is surprising, considering that the mean reproduction number also increases with *n*_*I*_. The reason for this counterintuitive result is that the probability of eventual extinction depends strongly on the probability that a cell produces zero secondary infections. This probability, P(R=0), is more sensitive to *θ* for smaller values of *n*_*I*_, which explains why the probability of extinction decays faster as a function of *θ* for smaller values of *n*_*I*_. Figure 9*b* shows how the probability of extinction starting with one infected cell depends on the number of infectious stages (and $\bar{R}$), for the fixed value of *θ* ≈ 0.59 which comes from the parameter values in table 3. For this value of *θ*, it can be seen that the probability of extinction is an increasing function of *n*_*I*_, even though the mean reproduction number $\bar{R}$ grows with *n*_*I*_. These results provide further evidence that focusing only on the mean reproduction number can be very misleading.

## 4. Discussion

The mean value of the cellular reproduction number has been computed making use of deterministic mathematical models for a number of viruses [28–31]. In some instances, these values have been compared between different viral strains as a measure of relative viral fitness [32]. Although the value of $\bar{R}$ is often used to compare viruses and predict the outcome of infection, this number alone might not be enough to do so. For example, in the early stages of viral infection, stochastic effects are important. In fact, data on the probability of recovery after acute HCV infection suggest that about 30% of infected people spontaneously clear infection and do not develop chronic infection [33]. When stochastic effects are considered, there is heterogeneity in the viral burst size, which is the number of viral progeny generated from an infected cell during its lifetime, as well as heterogeneity in the reproduction number, which is the number of secondary infections that a single infected cell produces. One can capture this heterogeneity by investigating the probability distributions of these two variables. Indeed, two viruses may have the same $\bar{R}$, but different reproduction number probability distributions, for instance, owing to differences in the lifespans of infected cells or the mechanism of virion release.

Here we have shown how to calculate the probability distributions of the random variables for the burst size and the reproduction number, for two stochastic viral dynamics models. We considered a model in which virions are released from infectious cells by budding at a constant rate. This model, shown in (2.1) and figure 1, includes an eclipse phase to represent the period after a virion infects a target cell, during which the viral genome is replicated but no virions are released. The duration of the eclipse phase follows an Erlang distribution, as does the time spent in the infectious phase until virus-induced cell death. The time until infected cells are killed by the immune system is assumed to follow an exponential distribution. Thus, the time to the death of an infected cell is the minimum of these two competing mechanisms. In addition to the model of viral release by budding, we also considered a model of viral release by bursting. This model assumes that viral particles are generated intracellularly at a constant rate and are eventually released in a burst event, but only if the infected cell is not killed by the immune system.

Given the burst size distribution, we have presented two methods to calculate the reproduction number distribution. The simpler method assumes that the target cell population remains constant (i.e. it is not depleted as these cells become infected). This assumption is valid if the system is well mixed and there is a large number of target cells, which could be the case early in infection. However, for some routes of infection, or for particular viruses, the availability of target cells may be limited. Thus, we introduced a different method that can be used in this instance, in which the number of target cells decreases due to infection events.

For *in vitro* scenarios, the effect of the immune system can be removed by setting *ν*_*E*_ = *ν*_*I*_ = 0 in the model, as in the models by Liao *et al.* [2] and Yan *et al.* [7], which were parametrized using *in vitro* experimental data. In the case where *ν*_*E*_ = *ν*_*I*_ = 0, we showed that the burst size and reproduction numbers follow negative binomial distributions. We can draw a comparison with the epidemic reproduction number, where gamma-distributed individual heterogeneity in the infection process results in a negative binomial distribution of secondary infections from each case [34]. The shape parameter of the negative binomial reproduction number distribution is sometimes called the *dispersion parameter* of the distribution, and it represents the degree of transmission heterogeneity, which arises from a broad range of biological and social factors that influence transmission. A low value of the dispersion parameter corresponds to a high level of dispersion in the distribution and suggests that a small number of infected individuals, known as ‘superspreaders’, may contribute to many secondary infections. The value of the dispersion parameter is particularly important for the early dynamics of an epidemic when there are only a few infected individuals. For example, Lloyd-Smith *et al.* [34] investigated the probability of stochastic extinction for an outbreak beginning with one infected individual, for different values of the dispersion parameter. Smaller values of the dispersion parameter were shown to increase the probability of stochastic extinction, but a small dispersion parameter also means that an epidemic can quickly take off due to the possibility of superspreading events, leading to infrequent but explosive epidemics. In the model studied here, the dispersion parameter of the negative binomial distribution in (2.11), given by *n*_*I*_, is an integer greater than or equal to 1, since it corresponds to the number of stages in the Erlang-distributed infectious period. However, heterogeneity in the within-host reproduction number can arise from a broad suite of factors other than the infectious period of the cell. Including other sources of variation into the model would impact the distribution of virions released from an infected cell. This could have consequences such as decreasing the predicted chance of successful infection establishment, or introducing the potential for superspreader cells.

For stochastic models of within-host viral dynamics, the distribution of the reproduction number can be used to calculate the probability that infection will become established in an individual, given an initial viral dose. We have shown how to calculate the probability of viral extinction for the budding and bursting models, and that the shape of the reproduction number distribution can have a significant impact on this probability. Previous studies of the probability of within-host viral extinction have focused on reproduction number distributions for which the means are the same in each case, but the shape of the distributions differ. This is similar to the case of epidemic models discussed above, where increased variability in individual infectiousness has been shown to increase the probability of stochastic extinction in an outbreak beginning with one infected individual [34]. For example, Pearson *et al.* [1] studied differences between continuous viral release and bursting, with the reproduction number being geometrically distributed in the continuous release case and assumed to either be a fixed value or Poisson distributed in the bursting case. By comparing the geometric reproduction number distribution and the Poisson distribution (with the same mean), Pearson *et al.* [1] showed that, with the greater variability of the geometric distribution, there was a larger chance of viral extinction and a lower probability of successful infection. Yuan and Allen [11] also considered a similar model to (2.1) but without an eclipse phase and with only one infectious stage. They studied the difference in the probability of viral extinction when comparing a model of the budding strategy with a geometrically distributed burst size, and the bursting strategy with a fixed burst size. They showed that the bursting strategy was more successful for viral invasion when a model without immune response was considered, but that the more successful strategy switched to budding when the immune response was included in the model. The results of both Pearson *et al.* and Yuan and Allen indicate that the distributions of the burst size and reproduction number are important in determining the probability of viral extinction; the mean alone is not enough. We have further shown that an increase in the expected reproduction number does not automatically imply a decrease in the probability of extinction, since the outcome depends on the distribution of the random variable.

In the electronic supplementary material, we studied a stochastic version of a previously published deterministic model of HCV infection in which the rate of virion release by budding is not constant, but depends on the intracellular viral dynamics [17]. The deterministic model by Guedj *et al.*, presented in equation (1) of the electronic supplementary material, is equivalent to a model in which the rate of viral release varies continuously over an infected cell’s lifespan. For the parameter values estimated by Guedj *et al.*, the release rate approaches a constant steady state fairly quickly, making the burst size and reproduction number distributions similar to the negative binomial distributions derived for the model of constant viral release by budding in the absence of immune response. However, the age-dependent viral release rate has a more noticeable impact when the infectious period follows an exponential distribution (*n*_*I*_ = 1), since cells that die very soon after infection release rather fewer virions than they would in the case of a constant release rate. The model for HCV considered here only includes simple intracellular dynamics; the model has a single equation for the intracellular viral genome, which increases by viral replication and is lost due to decay or release from the cell by budding. However, the method shown to calculate the burst size distribution for this particular model of HCV dynamics can be directly generalized to other models in which the release of virions from infected cells is a Poisson process and a functional form of the age-dependent viral release rate can be found. Furthermore, the method could be extended and implemented for models with more complex intracellular viral kinetics, such as those to describe co-infection and reassortment of segmented viruses.

A shortcoming of our approach is that we have focused only on stochastic models that have been derived from deterministic equations. *In vitro* experimental techniques that enable visualization of viral replication and virion release from single cells provide the opportunity for future work to focus on developing and parametrizing a much wider variety of stochastic models [35]. However, it will also be necessary to model the impact of *in vivo* processes that may not be observed *in vitro*, such as immune system clearance of infected cells. In §3.2, we showed that this can have important biological implications in terms of the likelihood of infection establishment. While it is challenging to directly measure immune system clearance experimentally, calibrating models with *in vivo* data can allow parameters that are not measurable *in vitro* to be estimated. For example, during treatment of chronic HCV infection, there is strong evidence of an exponential decay of infected cells [36,37], where this rapid death of infected cells is thought to be due to immune responses.

While the theory and examples presented here emphasize the importance of understanding reproduction number variation and modelling processes that contribute to this heterogeneity, there are important mechanisms that are not captured by the simple models presented here. For some viruses, such as HCV and respiratory syncytial virus, infected cells can directly infect target cells via cell–cell contacts [38–41]. Including this infection mechanism into the model may alter the predicted within-host reproduction number distribution. Furthermore, for the models presented here, variation in the reproduction number mainly results from variation in the infectious period of cells. However, there are many other aspects of host-cell biology that can make important contributions to heterogeneity of the within-host reproduction number. For example, cell-to-cell variation in the innate immune response (e.g. the response to type I interferons (IFN)) can lead to cell differences in the efficiency of viral transcription [42]. While in the models presented here, each cell is assumed to be infected with only one virion, cellular co-infection is common for many viral diseases. Thus, another source of variation across a heterogeneous infected cell population is the multiplicity of infection of individual cells, which can lead to heterogeneity in cell death rates and viral production rates [43]. Finally, cells that are in a certain phase of the cell cycle when they become infected may enable more efficient replication for some viruses [44].

In conclusion, computing the burst size and reproduction number probability distributions for stochastic viral dynamics models is a first step to compare these distributions for a range of viruses (or strains) and model structures. Furthermore, the reproduction number distribution can have surprising consequences for the probability of viral extinction. We have demonstrated this through an example in which the probability of viral extinction actually increases as the mean reproduction number increases.

## Data Availability

Computer codes (in Python) for reproducing the results in figures 3–9 are available from the Zenodo repository: https://doi.org/10.5281/zenodo.10391662 [45] and from the GitHub repository: https://github.com/Bevelynn/viral-reproduction-number [46]. Results for a mathematical model of HCV viral dynamics with an age-dependent viral release rate are provided in electronic supplementary material [47].
